# Worker health and well-being in Ontario’s electrical sector: a quantitative study of occupational health outcomes

**DOI:** 10.3389/fpubh.2025.1735294

**Published:** 2026-01-12

**Authors:** Donia Obeidat, Hong Ki Chloe Lau, Javier Mencia-Ledo, Sana Siddiqui, Avasa Sarawan, Zhiyang Shi, Raihana Premji, Aaron Howe, Ali Bani-Fatemi, Ali Asgary, Basem Gohar, Vijay Kumar Chattu, Geoffrey Maina, Thankam Sunil, Behdin Nowrouzi-Kia

**Affiliations:** 1 Department of Occupational Science and Occupational Therapy, Temerty Faculty of Medicine, University of Toronto, Toronto, ON, Canada; 2 Department of Rehabilitation Science, Faculty of Applied Medical Science, Jordan University of Science and Technology, Irbid, Jordan; 3 Disaster & Emergency Management, Faculty of Liberal Arts and Professional Studies, York University, Toronto, ON, Canada; 4 Department of Population Medicine, University of Guelph, Guelph, ON, Canada; 5 Centre for Research in Occupational Safety & Health, Laurentian University, Sudbury, ON, Canada; 6 Department of Public Health, Health Administration, Information, and Health Sciences, Tennessee State University, Nashville, TN, United States; 7 Central Asian Regional Center for Planetary Health, Semey Medical University, Semey, Kazakhstan; 8 Department of Community Medicine, Faculty of Medicine, Datta Meghe Institute of Medical Sciences (DMIMS), Wardha, India; 9 College of Nursing, University of Saskatchewan, Prince Albert, SK, Canada; 10 Department of Public Health, University of Tennessee, Knoxville, TN, United States; 11 Krembil Research Institute-University Health Network, Toronto, ON, Canada

**Keywords:** apprentices, burnout, Canada, electricians, musculoskeletal disorders, occupational health, psychological distress, sleep quality

## Abstract

**Introduction:**

In Canada, Ontario’s shortage of electricians is linked to high physical demands and psychosocial stressors that may undermine worker well-being.

**Methods:**

Using a Sustainable Development Goals (SDGs) informed lens, we conducted a cross-sectional online survey of self-employed electricians in Ontario (*n* = 188), assessing musculoskeletal symptoms (12-month), sleep quality, psychological distress, burnout (personal, work-related), and job satisfaction.

**Results:**

Overall, 90.2% reported at least one musculoskeletal symptom in the past 12 months. Regression analyses revealed that sleep quality and years of experience significantly predicted psychological distress and burnout, with poorer sleep linked to higher personal and work-related burnout, greater psychological distress, and lower job satisfaction. More years of experience were associated with lower distress and burnout. Women reported higher psychological distress than men, highlighting inequality and discrimination in male-dominated trades. Apprentices experienced greater psychological distress [8.96 (SD = 8.29)] than non-apprentices [4.99 (SD = 6.59)], reflecting vulnerabilities associated with low wages, job insecurity, and a lack of support.

**Discussion:**

These findings highlight the complex interplay of physical, psychological, and socio-structural factors in shaping electricians’ health. Targeted interventions are crucial for promoting sustainable and inclusive environments in the electrical industry.

## Introduction

Workers in the skilled construction trades play an essential role in the global economy, including Canada (1), the United Kingdom (2), and Australia (3), contributing to residential, commercial, industrial, and infrastructure projects. Within this sector, trade professionals, including bricklayers, carpenters, electricians, plumbers, welders, and heavy equipment operators, perform the hands-on tasks necessary to build and maintain physical structures. However, many of these workers, particularly electricians, face persistent challenges, including systemic disparities and widespread mental health concerns (4, 5). For example, the construction industry remains heavily male-dominated, with male workers comprising over 80% of the workforce (6). Furthermore, the skilled trades experiences elevated rates of mental ill-health and suicide compared to other sectors (5, 7, 8). In particular, 70% of construction industry workers report experiencing mental health issues like excessive stress (9). Commonly reported workplace stressors that contribute to mental health issues include work overload, poor working conditions, interpersonal conflict, and project role ambiguity (10). Long and irregular working hours, and frequently exceeding standard full-time loads, are associated with both adverse physical and mental health outcomes, including fatigue, sleep disruption, cardiovascular diseases, and burnout (7, 8, 11–13). Poor mental health is detrimental to worker well-being and is linked to increased risk of workplace injuries and fatalities (7, 14, 15). These issues are compounded by stigma surrounding mental health and limited access to support services (8, 16–18). Together, these challenges underscore the pressing need for systemic reforms and sustainable support to promote equity, improve mental health outcomes, and ensure the long-term viability of workers in the construction trades in Canada.

The skilled trades industry also has a high prevalence of musculoskeletal injuries, with studies reporting that approximately 50% of construction apprentices experienced musculoskeletal symptoms in the past 12 months (19, 20). These are injuries to the musculoskeletal system, including joints, ligaments, tendons, and muscles (21). Common musculoskeletal symptoms are frequently observed among apprentices in the skilled trades, including those affecting the wrists and hands, knees and feet, lower and upper back, neck and shoulders, elbows, hips, and thighs (22). Such injuries may occur due to excess strain on the body, commonly seen in physically demanding work. These patterns can be particularly harmful when repeated over extended periods and in poor working conditions (21).

Small and medium-sized enterprises (SMEs), which comprise a significant portion of Canada’s skilled trades sector, face additional constraints due to limited financial, time, and human resources (23). These barriers can hinder the implementation of mental health initiatives despite substantial psychological distress among SME owners/managers. For instance, 36.8% of 217 surveyed SME leaders reported high or very high levels of psychological distress, exceeding rates in the general working population (24). This distress is linked to increased rates of both absenteeism and presenteeism, often resulting in significant productivity losses when individuals continue to work while unwell. Despite these challenges, research suggests that psychological distress can be effectively reduced through brief, low-cost interventions, highlighting a promising avenue for mental health support within resource-limited SME environments (12).

Structural inequalities intensify these occupational health challenges. In Canada, representatives from the electrical industry reported that 90 to 100% of workers who work on power lines in the field are men (25). This can create many challenges in the workforce, including cultures that facilitate discrimination and hostility towards women (26). Moreover, men in male-dominated industries are less likely to seek mental health help, despite being more likely to experience depression and related mental health challenges (27, 28). Gender and ethnic disparities have also been shown to influence psychological well-being. Due to the prevalence of harassment and discrimination in construction, female workers in the industry are more likely to report higher stress, lower job satisfaction, and poorer work-life balance than men (5, 29). In addition, facing challenges such as language barriers, discrimination and wage disparities is another complication that migrant and racialized workers experience (5, 10, 30). Risk exposure also varies by role; tradespeople and laborers encounter greater physical demands and higher substance-use risk, whereas supervisory roles carry heavier psychological demands related to time pressure and interpersonal conflict (23, 24). Additionally, the cultural stigma associated with mental illness that is reinforced by traditional masculine norms continues to discourage help-seeking among construction workers (30).

In Ontario, Canada’s most populated province, the skilled trades sector, including the electrical sector, faces chronic labor shortages alongside high physical and psychosocial demands. These challenges are compounded by persistent gaps in diversity, equity, and inclusion, which negatively impact worker health and well-being. While past literature has examined the effects of various sociodemographic and socioeconomic variables on the physical and mental health of construction workers, there is a lack of literature on how these variables specifically affect the occupational health of electricians in Ontario’s electrical sector. Furthermore, past literature focuses on outcomes such as work engagement rather than physical and mental health outcomes. Musculoskeletal symptoms and sleep are significant factors to consider given their strong influence on physical and mental health outcomes in high-demand occupational contexts.

Given this backdrop, the current study aimed to examine the occupational health outcomes that impact the well-being of self-employed electrical workers. The specific objectives are (1) to assess disparities in mental and physical health outcomes among Ontario’s electricians focusing on musculoskeletal symptoms, psychological distress and personal burnout; (2) to evaluate the relationships between work burden, job satisfaction, and indicators of sustainable employment; (3) examining gender-specific differences in psychological distress and occupational health to assess equity gaps; and (4) examining apprenticeship status and its association with health and work outcomes.

## Materials and methods

### Research design and participants

This study employed a cross-sectional design to better understand the influence of various factors on the mental and physical health outcomes of Ontario’s electrical workers. Participants were recruited through our industry partner, the Ontario Electrical League (OEL). The OEL is a provincial non-profit organization that represents and supports professionals in Ontario’s electrical industry, including contractors, electricians, and manufacturers. The OEL distributed an e-mail script and invitation to potential participants. Individuals who expressed interest were invited to contact the research team for further information. A response rate could not be calculated, as the research team was removed from the recruitment process due to ethical reasons. A survey was developed and hosted on REDCap, a secure web-based platform for managing online surveys and databases. A link to the survey was then distributed via e-mail to self-employed, non-unionized electricians who are members of the OEL, and had provided informed consent to participate from January to March 2025. Eligibility criteria included being currently employed or self-employed as an electrician or electrical apprentice in Ontario and being at least 18 years of age. Individuals who work in administration, or those unable to complete the survey in English, were excluded. No names or direct personal identifiers were collected; data were stored on secure servers and analyzed in aggregate by the research team. The study was approved by the University of Toronto Research Ethics Board (Protocol #41194). The OEL had no access to individual-level data and did not influence analysis or interpretation.

### Survey measures

The Copenhagen Burnout Inventory (CBI) (31), the Nordic Musculoskeletal Questionnaire (32), the Pittsburgh Sleep Quality Index (PSQI) (33), the Kessler Psychological Distress Scale (K-6) (34), and the 12-Item Short-Form Health Survey (SF-12) (35). The CBI measures three main domains of burnout: personal burnout, work burnout, and colleague-related burnout (36), only personal and work burnout were included in the survey. The Nordic Musculoskeletal Questionnaire was used to identify areas of the body causing musculoskeletal problems (32). The K-6 was used to measure non-specific psychological distress in the general population (37). The PSQI evaluated sleep quality and disturbances in clinical populations within 1 month (38). PSQI scale indicates sleep quality, with a total score ranging from 0 to 21, where higher scores mean worse sleep. A global score greater than 5 is considered indicative of poor sleep quality, while scores of 5 or less suggest good sleep quality. The SF-12 is a general health questionnaire that has questions concerning both physical and mental health (39).

The CBI scores are positively associated with the degree of burnout, and a cutoff score of 50 indicates the presence of burnout (31). There is no consensus on a cutoff score for the Nordic Musculoskeletal Questionnaire as the tool is used to screen the prevalence of musculoskeletal pain in different body regions. Elevated scores on the K-6 suggest greater vulnerability to mental illness, with a score higher than 13 indicating moderate distress (37). The PSQI uses a cutoff score of 5, with higher scores corresponding to greater severity of sleep disturbances (38). Finally, job satisfaction was measured using a single 4-point Likert item asking for the overall job satisfaction. Please see Supplementary Table S1 for more details on the measures used and Supplementary Data Sheet 1 for the complete survey.

**Figure 1:**
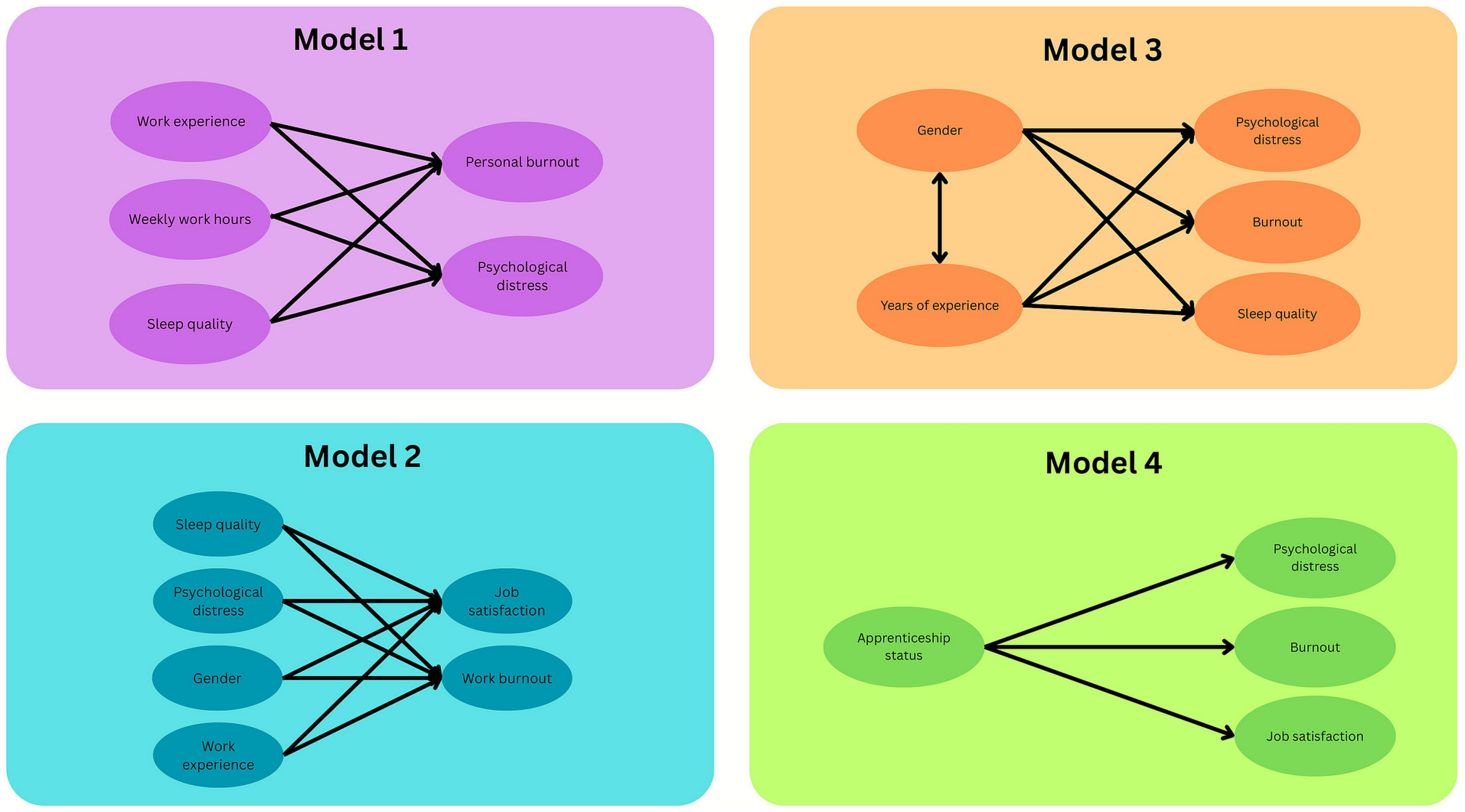
Overview of study models guided by SDGs.

The present study includes four prespecified models that examine how structural and individual factors relate to health, work burden, and equity outcomes (see Figure 1).

For the first model, NMQ results were presented as counts and percentages, and the model examined whether experience, weekly work hours, and sleep quality predicted psychological distress (K-6) and personal burnout (CBI). These variables were selected based on their established links to physical and mental strain in high-demand work settings. The model aimed to identify which factors contribute to elevated distress or exhaustion across the sample.

The second model focused on workload and job satisfaction. Outcomes included job satisfaction and work burnout (CBI). Predictors included sleep quality, psychological distress, gender, and experience. This model explored whether workers experiencing higher strain also reported lower satisfaction and whether specific job characteristics contributed to unsustainable workloads.

Gender-stratified models were run in the third model to assess whether the predictors of psychological distress, burnout, and sleep quality differed for men and women. Gendered-experience interaction terms were also included to test whether women apprentices experienced disproportionate strain. These analyses aimed to identify potential disparities within roles that may appear structurally equal but differ in their lived experiences.

The fourth model compared outcomes including distress, burnout, and job satisfaction between apprentices and non-apprentices, while adjusting for education and weekly hours. This model assessed whether those earlier in their training and career trajectory faced poorer outcomes, potentially reflecting financial or positional precarity.

### Data analysis

Analyses were conducted in R (version 4.4.2) for macOS (40). Continuous variables are reported as means and standard deviations (SD), while categorical variables are presented as frequencies and percentages. Complete case analysis was used since missing data only affected a few instances of just two variables. Prior to model building, multicollinearity among predictors was assessed using variance inflation factors (VIF). To examine the associations, a range of regression models were built depending on the nature of the response variable. For primary analyses, separate multiple linear regressions were fit for the primary outcomes: psychological distress, CBI-personal, and CBI-work burnout. Model fit for linear regression was evaluated using *R*^2^ values, adjusted *R*^2^, and examination of residual plots to verify assumptions of normality, homoscedasticity, and linearity. For logistic regression models, goodness of fit was assessed using classification accuracy. For primary analyses, separate multiple linear regressions were fit for the primary outcomes: psychological distress, CBI-personal, and CBI-work burnout. Prespecified predictors were years of experience, sleep quality (PSQI) and weekly hours, as well as K6 for some models. Job satisfaction was modelled using ordinal logistic regression, with PSQI, K6, years of experience, and gender as predictors. Nonetheless, if the proportional odds assumption was violated, multinomial logistic regression was used instead. For equity analyses (apprenticeship), apprentices were compared to licensed professionals based on K6/CBI/satisfaction scores, adjusting for education and weekly hours. Results are reported with 95% confidence intervals, and a two-tailed *p* < 0.05 was considered statistically significant.

## Results

A total of 188 electrical workers in Ontario completed the survey, yielding a completion rate of 82.2%. Most participants identified as male (89.9%) and were white (77%): the mean age was 34.5 (SD 12.7) years. Participants reported a mean 9.60 (SD = 9.35) years of electrical-sector experience and 41.2 (SD = 5.64) h of working duration/week. Regarding education, 113 (60%) participants had completed a college-level education, 50 (27%) had completed high school, and 15 (8.0%) had completed a university degree. More than half of the participants were apprentices (58%). The full demographic characteristics are shown in Table 1.

**Table 1:** Participants characteristics and working characteristics (*n* = 188).

Characteristic	Overall (*n* = 188)	Apprentices (*n* = 109)	Non-apprentices (*n* = 79)
Gender			
Man	169 (89.9%)	95 (87.2%)	74 (93.7%)
Woman	16 (8.6%)	12 (11%)	4 (5.1%)
Prefer not to answer	3 (1.6%)	2 (1.8%)	1 (1.3%)
Age			
Mean age	34.5 (12.7)	27.7 (7.3)	43.8 (12.6)
18–29	85 (45.5%)	73 (67.6%)	12 (15.2%)
30–45	63 (33.7%)	32 (29.6%)	31 (39.2%)
46–60	30 (16%)	3 (2.8%)	27 (34.2%)
>60	9 (4.8%)	0 (0.0%)	9 (11.4%)
Marital status			
Married	113 (60.8%)	52 (48.6%)	61 (77.2%)
Single	67 (36%)	55 (51.4%)	12 (15.2%)
Divorced	3 (1.6%)	0 (0.0%)	3 (3.8%)
Separated	3 (1.6%)	0 (0.0%)	3 (3.8%)
Ethnicity			
White	145 (77.1%)	77 (70.6%)	68 (86.1%)
Another ethnicity	43 (22.9%)	32 (29.4%)	11 (13.9%)
Education			
Incomplete high school	3 (1.6%)	2 (1.8%)	1 (1.3%)
Completed high school	51 (27.1%)	43 (39.4%)	8 (10.1%)
College	113 (60.1%)	54 (49.5%)	59 (74.7%)
University	15 (8.0%)	7 (6.4%)	8 (10.1%)
Other	6 (3.2%)	3 (2.7%)	3 (3.9%)
Average working hours per week	41.74 (6.27)	39.41 (4.68)	44.96 (6.77)
Working experience in electrical field*	10.21 (10.42)	4.35 (3.45)	18.14 (11.43)

Data presented as mean (SD)/*n* (%).

*Working experience reflects years worked to date in the electrical field, including time as an apprentice.

### Health outcomes

The sample exhibited mild levels of personal burnout (CBI domain average = 39.66, SD = 18.24) and work burnout (CBI domain average = 36.08, SD = 16.37), as well as regular levels of psychological distress (K6 average = 7.3, SD = 7.3). The SF-12 scores indicated that the sample had a consistently better-than-average health-related quality of life, with an overall average of 73.14 (SD = 14.17). This was observed in both mental health (average = 68.65, SD = 16.65) and physical health sub-scales (average = 78.94, SD = 16.34). Nonetheless, the average sleep quality score was 7.08 (SD = 3.33), reflecting poor sleep quality overall, with 127 participants (67.55% of the sample) reporting scores higher than 5. Table 2 shows the complete health outcome summary.

**Table 2:** Health outcome summary.

Outcome	Overall (*n* = 188)	Apprentices (*n* = 109)	Non-apprentices (*n* = 79)
Personal burnout (Copenhagen)	39.66 (18.24)	37.61 (20.26)	42.09 (19.29)
Work burnout (Copenhagen)	36.08 (16.37)	35.31 (18.26)	37.39 (17.1)
Psychological distress (K6)	7.3 (7.87)	8.96 (8.29)	4.99 (6.58)
Sleep quality (PSQI)	7.08 (3.33)	6.75 (3.25)	7.32 (3.38)
Health-related quality of life (SF-12)	73.14 (14.17)	75.56 (11.29)	71.39 (15.76)
Mental health sub-score	68.65 (16.56)	71.44 (13.39)	66.63 (18.32)
Physical health sub-score	78.94 (16.34)	81.29 (14.48)	77.24 (17.43)

Data presented as mean (SD).

Among the 188 participants, 90.4% reported musculoskeletal symptoms in at least one region of the body. The highest reported regions were the lower back and shoulder, prevalent in 58 and 55.6% of the participants, respectively. Remarkably, apprentices consistently showed higher prevalences of pain in almost all areas. Significant differences were observed in the neck, shoulder, and lower back, likely due to differences between the two groups in physical workload and strain. Table 3 displays the breakdown of musculoskeletal symptoms by body region and apprentice status.

**Table 3:** Prevalence of musculoskeletal symptoms by body region over the last 12 months.

Body region	Overall (*n* = 188)	Non-apprentices	Apprentices
Overall (MSP)	170 (90.4%)	72 (91.1%)	98 (89.9%)
Neck	**96 (51.1%)**	**35 (44.3%)**	**61 (56%)**
Shoulder	**104 (55.6%)**	**40 (51.3%)**	**64 (58.7%)**
Elbow	27 (14.4%)	6 (7.6%)	21 (19.3%)
Wrist or hands	81 (43.3%)	32 (40.5%)	49 (45.4%)
Upper back	58 (30.9%)	16 (20.3%)	42 (38.5%)
Lower back	**109 (58%)**	**40 (50.6%)**	**69 (63.3%)**
Hips	35 (18.6%)	15 (19%)	20 (18.3%)
Knees	70 (37.2%)	25 (31.6%)	45 (41.3%)
Ankles or feet	30 (16%)	13 (16.5%)	17 (15.6%)

Prevalence of pain in each area, stratified by apprentice status.

Bold rows indicate statistically significant differences.

Table 4 presents the results of multiple regression analyses predicting psychological distress, and burnout. The findings consistently identify sleep quality and professional experience as key predictors, while weekly work hours was not statistically significant in any model. Specifically, the regression for psychological distress (K-6) yielded a statistically significant model (adjusted *R*^2^ = 0.174, *F* = 13.94, *p* < 0.001). Poorer sleep quality was a strong positive predictor of higher distress (*β* = 0.75, 95% CI: [0.45–1.06], *p* < 0.001), whereas a greater number of years of experience significantly predicted lower distress (*β* = −0.19, 95% CI: [−0.29, −0.08], *p* = 0.001).

**Table 4:** Multiple linear regression of psychological distress and burnout (personal and work).

Predictor	Psychological distress (K6)			Personal burnout			Work burnout		
	*β*	95% CI	*p*	*β*	95% CI	*p*	*β*	95% CI	*p*
(Intercept)	6.14	−1.23–13.52	0.102	23.50	3.82–43.19	0.020	14.76	−2.86–32.38	0.100
PSQI (sleep quality)	0.75	0.45–1.06	<0.001	1.63	0.82–2.44	<0.001	1.26	0.49–2.03	0.001
Experience	−0.19	−0.29–−0.08	0.001	−0.39	−0.67–−0.11	0.006	−0.27	−0.53–−0.01	0.041
Weekly hours	−0.06	−0.23–0.11	0.497	0.22	−0.25–0.68	0.356	0.33	−0.08–0.75	0.111
K6 distress							0.23	−0.21–−0.64	0.195

CBI: Copenhagen Burnout Inventory. Experience is years worked and weekly hours is average hours per week.

A similar pattern emerged for personal burnout, (adjusted *R*^2^ = 0.103, *F* = 23.50, *p* < 0.001). Again, poorer sleep quality was associated with increased burnout (*β* = 1.63, 95% CI: [0.82, 2.44], *p* < 0.001), while more years of experience served as a protective factor (*β* = −0.39, 95% CI: [−0.67, −0.11], *p* = 0.006). For work-related burnout, a regression model including interactions was significant (*R*^2^ = 0.137, *F* = 6.061, *p* < 0.001). The results confirmed the consistent trends: poorer sleep quality was a significant positive predictor (*β* = 1.43, 95% CI: [0.71, 2.15], *p* < 0.001), and greater experience was a significant negative predictor (*β* = −0.27, 95% CI: [−0.53, −0.01], *p* = 0.041). Finally, a multinomial logistic regression examined predictors of job satisfaction (Nagelkerke *R*^2^ = 0.113). In this model, sleep quality was the sole significant predictor, with poorer sleep associated with higher odds of job dissatisfaction (OR = 1.13, 95% CI: [1.03, 1.25], *p* = 0.014).

Table 5 presents the results of the multiple linear regression analysis examining the impact of gender and years of experience on the K-6. The overall model explained 13.7% of the variance (adjusted *R*^2^ = 0.113, *F* = 8.20, *p* < 0.001). The results indicated that women experienced a significantly higher level of psychological distress than men (*β* = 8.12, 95% CI: [2.63, 13.61] *p* = 0.004), although the small number of women in the sample may have influenced this. Moreover, having worked more years of experience was associated with a significant reduction in psychological distress (*β* = −0.17, 95% CI: [−0.27, −0.06], *p* = 0.002). However, the interaction between gender and years of experience was not significant.

**Table 5:** Multiple linear regression of psychological distress.

Predictor	Kessler-6 (Psychological distress)		
	*β*	95% CI	*p*
(Intercept)	8.20	6.61–9.78	**<0.001**
Gender			
Men	1.00		
Women	8.12	2.63–13.61	**0.041**
Other	5.72	−20.90–32.34	0.672
Experience	−0.17	−0.27–−0.06	**0.002**
Gender [women] × experience	−0.46	−1.52–0.59	0.388
Gender [other] × experience	−0.08	−5.14–4.97	0.975

Model showing the interaction between years as electrician and gender, using men as the reference group. Experience is years worked and weekly hours is average hours z. Bold rows indicate statistically significant differences.

A follow-up t-test was conducted to compare psychological distress between genders. The mean difference in psychological distress between men and women was 8.25, and this difference was statistically significant (*t*-test, *p* = 0.004). Men reported a mean K-6 score of 6.5 (SD = 7.36), whereas women reported a substantially higher mean of 14.75 (SD = 9.49); participants who preferred not to disclose their gender had a mean score of 12.67 (SD = 0.58).

An additional model (Supplementary Table S1) testing interactions between apprenticeship and psychological distress while controlling for working hours/week and education was statistically significant (*r*^2^ = 0.084, *F* = 2.754, *p* = 0.014). Apprentice status was a significant positive predictor of psychological distress, indicating that apprentices had a higher level of psychological distress than non-apprentices (*β* = 3.49, 95% CI = 0.86–6.13, *p* = 0.010).

A comparison between apprentices and non-apprentices showed a statistically significant difference in psychological distress (K-6 scores) (*p* < 0.05). The mean K-6 differences between groups were 3.98. Apprentices’ mean K-6 scores were 8.96 (SD = 8.29), compared to non-apprentices’ mean of 4.99 (SD = 6.59).

The models for gender and years of experience on personal burnout, work burnout, and sleep quality, respectively, showed worse fits, explaining less than 5% of the variance. Nonetheless, they still consistently showed that more years of experienced improved scores, but that women had lower burnout scores than men.

## Discussion

The study adds sector-specific evidence on the well-being of electricians in Ontario. Among 188 participants, most identified as men and White, reflecting the characteristics of the Canadian electrical industry, which remains predominantly white and male-dominated. These composition details are important for interpreting generalizability. Furthermore, apprentices, electricians with less experience, and women showed a higher psychological distress compared to experienced electricians and men in the electrical industry.

This research found that electricians commonly experience Work-related Musculoskeletal Disorder symptoms in areas including the lower back, shoulders, and neck. These findings are consistent with previous studies, which indicate that skilled trades workers mainly reported symptoms affecting the neck, shoulders, and back (19, 22, 41, 42). Previous studies underscored MSDs as one of the most common occupational health problems in the skilled trades sector globally (43). Although not explored in our study, past literature has found that several occupational factors may contribute to the widespread prevalence of work-related musculoskeletal symptoms, including repetitive movements, forceful exertion, awkward postures, heavy lifting, and overhead work (43). The current study also found a higher prevalence of musculoskeletal symptoms among apprentices as compared to non-apprentice workers. This finding is strongly supported in the literature, with Rosecrance et al. (2001) reporting musculoskeletal symptoms in more than 50% of apprentices within the last 12 months (19). Additionally, Merlino et al. (2003) found similar rates of MSD symptoms in the neck (31.85%), shoulder (27.9%), and most commonly in the lower back (54.4%) (22). Our current findings, along with past research, provides some insight into how the injury management of electrical workers may be further improved, particularly in terms of lower back, shoulder, and neck injuries. The present findings revealed that while average weekly working hours, years of experience, and sleep quality do not explain a significant portion of variance in psychological distress in electricians, the workers’ years of experience and sleep quality were significantly correlated to psychological distress. This finding aligns somewhat with past studies, which indicate that years of work experience and sleep quality are substantial predictors of psychological distress among construction workers (44, 45). The significant relationship between years of experience and personal burnout may be because, as they spend more time in their field, they learn how to manage their stressors better and develop stronger coping strategies and in turn strengthen their stress resistance (46, 47). However, regarding weekly working hours, this finding is inconsistent with past research, which found that longer working hours were significantly and positively associated with psychological distress (48). The results of our study may contribute to the ongoing conversation on distress in electrical workers, and the inconsistency between our results and past literature prompts other researchers to further investigate the relationship between these variables and verify our findings.

Additionally, our study found that there is a significant and negative link between years of experience and personal burnout, and conversely, a correlation between poor sleep quality and personal burnout. Our findings are consistent with past literature, which shows the same relationship among electrical workers (45). Our study is novel in its investigation of the relationship between sleep quality and personal burnout in electrical workers, as there is limited literature on the relationship between sleep quality and burnout in this population, though insufficient sleep has been found to correlate with burnout in a general worker population (49–51). Our results also indicated that average weekly working hours is not significantly associated with personal burnout. Though, this finding contrasts with previous research, which has consistently identified extended working hours as a key predictor of work–life conflict among construction employees (52). The inconsistency may be speculated to relate to the fact that some prior studies examined overall burnout rather than personal burnout specifically (49–51). Further research should separately examine whether working hours have a differential influence on various types of burnout among electrical workers.

Moreover, burnout is typically categorized into three categories: emotional exhaustion, depersonalization, and a sense of reduced accomplishment, where individuals feel a lack of productivity and effectiveness (53, 54). It would be beneficial for future research to examine which dimension is influencing the outcomes. Interestingly, years of experience have been found not to significantly influence burnout in early-career construction management professionals in the United States, which somewhat contradicts our findings. The discrepancy may be due to the difference in the focus population, as our study only includes electrical workers. There is some likelihood that the effect years of experience have on personal burnout is only prominent in the electrical sector rather than the construction industry as a whole. Regardless, our study adds interesting insights to the exploration of predictors of personal burnout among Canadian electrical workers and lays the foundation for further research on personal burnout in this population.

This study found that women experienced a significantly higher level of psychological distress than men. These findings align with present literature, as previous studies have found similar results, with women facing considerably higher levels of psychological distress than men (55, 56). When speculating explanations, there are many potential ideas present in the literature. One study found that women in general were more likely to experience psychological distress when they are experiencing work-to-family conflict (55). In one qualitative study, women in construction cited that they experienced high levels of psychological distress due to multiple reasons, including dangerous work environments, gender discrimination, inadequate safety and protective equipment and a fear of negative consequences if they reported concerns (26). Moreover, these findings may also be a result of differences in reporting styles among sexes. Men tend to underreport mental health issues, with mental health service utilization being almost double for women in North America (57). However, this study had a small sample size of women. This may have facilitated more pronounced differences in psychological distress to appear between sexes. More research is needed to understand the specific relationship between differences in work environments and psychological distress experienced by women and men. Additionally, this study found no significant gender differences in years of experience or psychological distress. Although there is limited research examining this topic, one study found that work experience significantly predicted job autonomy, with no significant differences observed between women and men (44). This might mean that women and men experience similar instances of job stability as work experience increases, lowering the likelihood of experiencing psychological distress. Overall, our study helps contribute a novel finding that is limitedly explored in the construction electrical industry. Further research related to cause of the lack of significant gender differences in years of experience and psychological distress is needed to validate the findings of this study.

Our study further highlights that apprenticeship status is significantly associated with psychological distress among electricians, even though the overall model was not statistically significant. These findings are consistent with a growing body of research indicating that apprentices commonly experience elevated levels of psychological distress. Electrical sector apprentices reported higher psychological strain levels compared to workers and contractors in the same sector (45). Although speculative, many factors may contribute to these elevated levels. For instance, apprentices often suffer from long working hours, low wages, job insecurity, poor educational quality, and unrealistic expectations (58, 59). A further major contributor from the literature is workplace bullying, as well as a lack of general support. Bullying among construction industry apprentices is strongly associated with poor mental health outcomes, including greater psychological distress (60, 61). Similarly, the hierarchical structure and inadequate workplace support from workers were shown to exacerbate this effect and ultimately lead to an increase in vulnerability to distress (58). Lastly, practice readiness may play a role. Previous studies have found that the level of self-efficacy one has at work can predict the level of burnout experienced by workers (62). Moreover, one qualitative study found that the lack of work readiness in new graduate nurses creates aspects of psychological stress (63). More research is needed on electrician apprentices specifically to discover potential explanations for our findings.

Our study found that poorer sleep quality was associated with increased work-related burnout and job dissatisfaction. These outcomes are consistent with the emerging body of research in the construction sector, which demonstrates that poor sleep has a strong adverse effect on occupational health outcomes. One study found that decreased sleep quality is reported to influence job satisfaction through a serial mediation model (64). Moreover, Wendimu and Meshesha (2023) found that a large proportion of the construction worker sample experienced poor sleep quality, which was associated with low job satisfaction (65). Another study found that sleep difficulties were associated with elevated levels of work-related burnout (66). Among electricians specifically, work burnout was also correlated with sleep disturbances (45). Additionally, it has been found that among working individuals, sleep problems typically occur before low back pain and burnout symptoms (49). Chattu et al. in their research have concluded that sleep quality assessments are early risk indicators that help to reduce the incidence of wider range of morbidities (67). Therefore, this could demonstrate the need for sleep quality evaluations to be emphasized in health promotion programs as a crucial early risk indicator.

Taken together, this study created and used models that can align with the actions that advance the targets of SDGs 1, 3, 5, 8, 9, 10, and 11. Our findings align with SDG 3 targets by identifying risk factors for occupational ill-health, such as psychological distress and burnout, which can inform targeted workplace interventions to support healthier work lives for electrical workers. Within the framework of SDG 8 and 9, the findings of this study suggest that improving sleep conditions is integral to ensuring work is not only safe but also sustainable, in terms of electricians’ well-being and satisfaction. Work burnout and job satisfaction have a significant impact on retention, productivity, safety, and the overall quality of work.

Furthermore, our findings may inform the development of strategies catered to SDG 5 and 10. There is a need to develop workplace interventions that address the diverse lived experiences faced by various groups, including women. This will help ensure that workplace policies and practices support gender equality (SDG 5) and reduce inequality for marginalized groups (SDG 10). Lastly, our findings relate to SDG 1 and 11. Using apprenticeship status as a proxy of socioeconomic status, our findings suggest that socioeconomic conditions are linked to apprentices’ psychological distress including receiving lower wages, less schedule stability, and less support. Poor mental health can challenge apprentices’ ability to maintain stable employment. If apprentices are distressed and mentally unwell, their retention, performance, and satisfaction may suffer, which in turn may affect poverty reduction goals and impact broader community stability and sustainability. There is a need for workplace culture interventions to address psychosocial risk factors, including but not limited to fairness of treatment, support from supervisors and older colleagues, workload management, and clarity of expectations, to reduce distress among apprentices. Overall, by incorporating the SDGs to inform the study models, it provides a unique framework to understanding the occupational health of Canadian electrical workers and connects it to broader global development goals, highlighting the interconnectedness between personal well-being of workers and global sustainability.

### Limitations and future directions

Several limitations to this study should be considered. First, the data comes from a single cross-sectional survey of non-union, self-employed electricians from the Ontario Electrical League, that is, a single association. This may introduce selection bias which limits the generalizability and representativeness of the findings. Secondly, given that it was self-reported, the results may be influenced by recall bias and social desirability. Additionally, the causal relationship cannot be confirmed, as is often the case with most cross-sectional studies. Job satisfaction was assessed using a single item on a four-point scale, which limits how these results can be extrapolated to other studies using validated scales. The fact that musculoskeletal pain symptoms were only reported as prevalences limits interpretations on how they affect function. Additionally, all data were collected exclusively by the OEL, and our research team had no direct involvement in the data-collection process. Due to this reason, the number of individuals who were approached for this study is unknown, and we are unable to calculate a response rate. This introduces the potential for selection bias in our findings. Finally, the small sample size of participants may not be sufficient to detect differences between groups, specifically gender and other minority groups. Given the number of models that were fit on this limited sample, a multiple testing issue may arise. Consequently, we suggest that future studies include a larger sample size with diverse characteristics and employ a more prospective approach to replicate the preliminary findings reported in this research study.

## Conclusion

Framed within the SDGs, this study offers important insights into the health and well-being of electricians within the Canadian electrical sector. Work-related musculoskeletal disorder symptoms remain a highly prevalent health issue, particularly affecting the lower back, shoulders, and neck, underscoring the need for further preventive strategies, ergonomic assessments, and targeted interventions. Poor sleep consistently emerged as a predictor of burnout and job dissatisfaction; these findings emphasize the importance of recovery and rest in maintaining the well-being, productivity, and safety of electricians. This helps to achieve the goals of decent work, economic growth, and industry, innovation, and infrastructure (SDG 8 and 9).

Although there was a small number of women in the sample, this study found that women were more likely than men to report psychological distress. This might indicate the need for further research investigating the challenges for individuals of varying genders. Our work emphasizes the imperative for workplace policies and interventions that address gendered experiences and promote inclusivity, in line with the objectives of gender equality and reduced inequalities (SDGs 5 and 10). Moreover, apprentices presented greater psychological distress compared to non-apprentices, suggestive of vulnerabilities linked to inadequate wages, job instability, workplace harassment, and insufficient support. Consequently, there is a necessity for focused workplace interventions that enhance apprenticeship programs and foster supportive workplace cultures, thereby promoting the goals of poverty alleviation and sustainable community development (SDGs 1 and 11).

This research demonstrates that various physical, psychological, and social factors interact to impact the health and well-being of electricians. To address these issues, we recommend working on developing plans that incorporate improved ergonomics, enhanced sleep and fatigue management, psychosocial support, equitable policies for both men and women, and fair apprenticeship structures. The electrical sector can help create healthier and more sustainable work environments while also contributing to the achievement of SDGs.

## Data Availability

The raw data supporting the conclusions of this article will be made available by the authors, without undue reservation.
