# Supplementary material for: Worker health and well-being in Ontario’s electrical sector: a quantitative study of occupational health outcomes
Source: Front Public Health. 2026 Jan 12;13:1735294. doi: 10.3389/fpubh.2025.1735294 (PMC12833060; doi:10.3389/fpubh.2025.1735294)
Supplement: Supplementary file 1 [file Table_1.docx]

**Description of Measures**

| Scale | Description | Scoring | Psychometric properties |
| --- | --- | --- | --- |
| The Copenhagen Burnout Inventory (CBI) | The CBI measures burnout across three subscales: (1) personal burnout; (2) work-related burnout; and (3) client-related burnout. For this survey, only the personal and work-related burnout subscales were used (Kristensen et al., 2005). The measure contains 19 items rated on a five-point Likert-scale. | Scores on the CBI range from 0 to 100, with higher scores suggesting the presence of burnout (Kristensen et al., 2005). Scores that are 50 or higher across the three scales are indicative of a high level of burnout. | The CBI has been shown to have great internal consistency (Cronbach’s α=0.85- 0.94) and good validity across studies (Aiello et al., 2022; Bolatov et al., 2021; Kristensen et al., 2005; Shoman et al., 2021; Tran et al., 2023). |
| Nordic Musculoskeletal Questionnaire (NMQ) | The NMQ assesses musculoskeletal problems across 9 areas of the body: (1) neck; (2) shoulders; (3) upper back; (4) elbows; (5) low back; (6) wrist/hands; (7) hip/thighs; (8) knees; and (9) ankles/feet (Crawford, 2007). It includes forced choice yes or no items. | There is no total scoring for the NMQ (Crawford, 2007). An answer of “yes” indicates the prevalence of a musculoskeletal problem across one of the 9 regions of the body. | When testing the test-retest reliability of the tool, answers ranged from 0 to 23% (Crawford, 2007). When testing validity, there was a span of 0 to 20% disagreement. This is considered sufficient for a screening instrument. |
| Pittsburgh Sleep Quality Index (PSQI) | The PSQI is designed to estimate sleep quality and disturbance over a month (Buysse et al., 1989). The questionnaire employs both Likert-type and open-ended questions and converts the open-ended responses into scale scores based on given guidelines (Shahid et al., 2011). | Each question is scored from 0 to 3, with higher scores representing more severe sleep disturbances (University of Pittsburgh, n.d.). The global score can range from 0-21, with scores greater than 5 representing poor sleep quality (University of Pittsburgh, n.d.). | The PSQI has been shown to demonstrate good construct validity and internal consistent reliability (Cronbach's α = 0.80 across groups) (Carpenter & Andrykowski, 1998). |
| Kessler Psychological Distress Scale (K-6) | The K-6 is a short form of K-10, a measure that examines non-specific psychological distress (Kessler et al., 2002). The measure has a total of 6 questions, with each response rated on a 5-point Likert scale (Kessler et al., 2002). | The total score can range from 6 to 30, with higher scores indicating higher severity of psychological distress (Kessler et al., 2003). Scores greater than 13 indicate severe mental illness (Kessler et al., 2003). | The K-6 has been shown to have great internal consistency reliability (α = .89) (Kessler et al., 2002). It also significantly and strongly predicts several mental disorders (Umucu et al., 2022). |
| 12-Item Short-Form Health Survey (SF-12) | The SF-12 is a short form of 36-Item Short-Form Health Survey (SF-36), a measure that assesses both physical and mental health conditions (Lim & Fisher, 1999). The measure has 12 total questions, with some using a 6-point Likert scale and others using a binary (yes/no) scale (Ohrnberger et al., 2020). | The physical and mental components of the SF-12 are scored separately, with scores ranging from 0-100 (Ware et al., 1998). While there is no formal cutoff score, scores above 50 generally indicate better-than-average health-related quality of life (Physiopedia, n.d.). | The SF-12 demonstrated good construct validity (Lim & Fisher, 1999). When compared to the 36-item Short Form scale, the 12-item Physical Component Summary was shown to have relative validity ranging from 0.43 to 0.93 (median = 0.67), while the 12-item Mental Component Summary demonstrated relative validity from 0.60 to 1.07 (median = 0.97) (Ware et al., 1996). |

**References**

Aiello, E. N., Fiabane, E., Margheritti, S., Magnone, S., Bolognini, N., Miglioretti, M., & Giorgi, I. (2022). Psychometric properties of the Copenhagen Burnout Inventory (CBI) in Italian Physicians. *La Medicina Del Lavoro*, *113*(4), e2022037. https://doi.org/10.23749/mdl.v113i4.13219

Bolatov, A. K., Seisembekov, T. Z., Askarova, A. Zh., Igenbayeva, B., Smailova, D. S., & Hosseini, H. (2021). Psychometric Properties of the Copenhagen Burnout Inventory in a Sample of Medical Students in Kazakhstan. *Psychology in Russia: State of the Art*, *14*(2), 15–24. https://doi.org/10.11621/pir.2021.0202

Buysse, D. J., Reynolds, C. F., Monk, T. H., Berman, S. R., & Kupfer, D. J. (1989). The Pittsburgh sleep quality index: A new instrument for psychiatric practice and research. *Psychiatry Research*, *28*(2), 193–213. https://doi.org/10.1016/0165-1781(89)90047-4

Carpenter, J. S., & Andrykowski, M. A. (1998). Psychometric evaluation of the pittsburgh sleep quality index. *Journal of Psychosomatic Research*, *45*(1), 5–13. https://doi.org/10.1016/S0022-3999(97)00298-5

Crawford, J. O. (2007). The Nordic Musculoskeletal Questionnaire. *Occupational Medicine*, *57*(4), 300–301. https://doi.org/10.1093/occmed/kqm036

Kessler, R. C., Andrews, G., Colpe, L. J., Hiripi, E., Mroczek, D. K., Normand, S.-L. T., Walters, E. E., & Zaslavsky, A. M. (2002). Short screening scales to monitor population prevalences and trends in non-specific psychological distress. *Psychological Medicine*, *32*(6), 959–976. https://doi.org/10.1017/S0033291702006074

Kessler, R. C., Barker, P. R., Colpe, L. J., Epstein, J. F., Gfroerer, J. C., Hiripi, E., Howes, M. J., Normand, S.-L. T., Manderscheid, R. W., Walters, E. E., & Zaslavsky, A. M. (2003). Screening for Serious Mental Illness in the General Population. *Archives of General Psychiatry*, *60*(2), 184–189. https://doi.org/10.1001/archpsyc.60.2.184

Kristensen, T. S., Borritz, M., Villadsen, E., & Christensen, K. B. (2005). The Copenhagen Burnout Inventory: A new tool for the assessment of burnout. *Work & Stress*, *19*(3), 192–207. https://doi.org/10.1080/02678370500297720

Lim, L. L.-Y., & Fisher, J. D. (1999). Use of the 12-item Short-Form (SF-12) Health Survey in an Australian heart and stroke population. *Quality of Life Research*, *8*(1–2), 1–8. https://doi.org/10.1023/A:1026409226544

Ohrnberger, J., Anselmi, L., Fichera, E., & Sutton, M. (2020). Validation of the SF12 mental and physical health measure for the population from a low-income country in sub-Saharan Africa. *Health and Quality of Life Outcomes*, *18*, 78. https://doi.org/10.1186/s12955-020-01323-1

Physiopedia. (n.d.). *12-Item Short Form Survey (SF-12)*. Physiopedia. Retrieved October 27, 2025, from https://www.physio-pedia.com/12-Item_Short_Form_Survey_(SF-12)

Shahid, A., Wilkinson, K., Marcu, S., & Shapiro, C. M. (2011). Pittsburgh Sleep Quality Index (PSQI). In A. Shahid, K. Wilkinson, S. Marcu, & C. M. Shapiro (Eds.), *STOP, THAT and One Hundred Other Sleep Scales* (pp. 279–283). Springer New York. https://doi.org/10.1007/978-1-4419-9893-4_67

Shoman, Y., Marca, S. C., Bianchi, R., Godderis, L., Van Der Molen, H. F., & Guseva Canu, I. (2021). Psychometric properties of burnout measures: A systematic review. *Epidemiology and Psychiatric Sciences*, *30*, e8. https://doi.org/10.1017/S2045796020001134

Tran, T. T. T., Nguyen, Q. T., Nguyen, N. T., Vu, S. T., Kaewboonchoo, O., Kawakami, N., & Nguyen, H. T. (2023). Psychometric properties and factor structure of the Vietnamese Copenhagen Burnout Inventory. *Journal of Occupational Health*, *65*(1), e12425. https://doi.org/10.1002/1348-9585.12425

Umucu, E., Fortuna, K., Jung, H., Bialunska, A., Lee, B., Mangadu, T., Storm, M., Ergun, G., Mozer, D. A., & Brooks, J. (2022). A National Study to Assess Validity and Psychometrics of the Short Kessler Psychological Distress Scale (K6). *Rehabilitation Counseling Bulletin*, *65*(2), 140–149. https://doi.org/10.1177/00343552211043261

University of Pittsburgh. (n.d.). *The Pittsburgh Sleep Quality Index (PSQI)*. https://www.sleep.pitt.edu/psqi

Ware, J. E., Kosinski, M., & Keller, S. D. (1996). A 12-Item Short-Form Health Survey: Construction of Scales and Preliminary Tests of Reliability and Validity. *Medical Care*, *34*(3), 220–233. https://doi.org/10.1097/00005650-199603000-00003

Ware, J. E., Kosinski, M., & Keller, S. D. (1998). *SF-12: How to Score the SF-12 Physical and Mental Health Summary Scales*. ResearchGate. https://www.researchgate.net/publication/242636950_SF-12_How_to_Score_the_SF-12_Physical_and_Mental_Health_Summary_Scales
