# Supplementary material for: Worker health and well-being in Ontario’s electrical sector: a quantitative study of occupational health outcomes
Source: Front Public Health. 2026 Jan 12;13:1735294. doi: 10.3389/fpubh.2025.1735294 (PMC12833060; doi:10.3389/fpubh.2025.1735294)
Supplement: Supplementary file 2 [file Table_2.docx]

**List of United Nations (UN) Sustainable Development Goals (SDGs)**

| **Goal** | **Description (Quoted Directly from United Nations (n.d.))** |
| --- | --- |
| 1: No Poverty | End poverty in all its forms everywhere |
| 2: Zero Hunger | End hunger, achieve food security and improved nutrition and promote sustainable agriculture |
| 3: Good Health and Well-Being | Ensure healthy lives and promote well-being for all at all ages |
| 4: Quality Education | Ensure inclusive and equitable quality education and promote lifelong learning opportunities for all |
| 5: Gender Equality | Achieve gender equality and empower all women and girls |
| 6: Clean Water and Sanitation | Ensure availability and sustainable management of water and sanitation for all |
| 7: Affordable and Clean Energy | Ensure access to affordable, reliable, sustainable and modern energy for all |
| 8. Decent Work and Economic Growth | Promote sustained, inclusive and sustainable economic growth, full and productive employment and decent work for all |
| 9. Industry, Innovation and Infrastructure | Build resilient infrastructure, promote inclusive and sustainable industrialization and foster innovation |
| 10. Reduced Inequalities | Reduce inequality within and among countries |
| 11. Sustainable Cities and Communities | Make cities and human settlements inclusive, safe, resilient and sustainable |
| 12. Responsible Consumption and Production | Ensure sustainable consumption and production patterns |
| 13. Climate Action | Take urgent action to combat climate change and its impacts |
| 14. Life Below Water | Conserve and sustainably use the oceans, seas and marine resources for sustainable development |
| 15. Life on Land | Protect, restore and promote sustainable use of terrestrial ecosystems, sustainably manage forests, combat desertification, and halt and reverse land degradation and halt biodiversity loss |
| 16. Peace, Justice and Strong Institutions | Promote peaceful and inclusive societies for sustainable development, provide access to justice for all and build effective, accountable and inclusive institutions at all levels |
| 17. Partnerships for the Goals | Strengthen the means of implementation and revitalize the Global Partnership for Sustainable Development |

***Note:*** Highlighted SDGs are related to this paper

**References**

United Nations. (n.d.). *THE 17 GOALS | Department of Economic and Social Affairs- Sustainable Development*. Retrieved October 7, 2025, from <https://sdgs.un.org/goals>
