# Supplementary material for: Worker health and well-being in Ontario’s electrical sector: a quantitative study of occupational health outcomes
Source: Front Public Health. 2026 Jan 12;13:1735294. doi: 10.3389/fpubh.2025.1735294 (PMC12833060; doi:10.3389/fpubh.2025.1735294)
Supplement: Supplementary file 3 [file Table_3.docx]

**Table S3.** Linear Regression of the Interaction Between Apprenticeship Status, Education, and Psychological Distress.

| **Kessler-6 (Psychological distress)** | | | | | |
| --- | --- | --- | --- | --- | --- |
| **Predictor** | | | **b** | **95% CI** | **p** |
| (Intercept) | | | 8.76 | -0.45 – 17.96 | 0.062 |
| Avg hours worked per week | -0.09 | -0.29 – 0.11 | 0.376 | | |
| Education [Completed high  school] | 0.34 | -2.35 – 3.03 | 0.801 | | |
| Education [Incomplete  high school] | -4.22 | -13.06 – 4.62 | 0.347 | | |
| Education [University] | 0.91 | -3.30 – 5.12 | 0.671 | | |
| Education [Other please  specify] | 4.89 | -1.44 – 11.21 | 0.129 | | |
| Apprentice | 3.49 | 0.86 – 6.13 | **0.010** | | |
