# Supplementary material for: Worker health and well-being in Ontario’s electrical sector: a quantitative study of occupational health outcomes
Source: Front Public Health. 2026 Jan 12;13:1735294. doi: 10.3389/fpubh.2025.1735294 (PMC12833060; doi:10.3389/fpubh.2025.1735294)
Supplement: Supplementary file 4 [file Data_Sheet_1.pdf]

Wired for Wellness, Investigating Electricians' Physical and Psychological Health: A Mixed-Methods Study

What is your gender?

- ◯ Man
- ◯ Woman
- ◯ Other, please specify
- ◯ Prefer not to answer

____

What is your marital status?

- ◯ Single
- ◯ Married/Common-Law
- ◯ Separated
- ◯ Divorced
- ◯ Widowed

What is your ethnicity?

- ☐ Black (e.g., African, African Canadian, Afro-Caribbean descent)
- ☐ East Asian (e.g., Chinese, Japanese, Korean, Taiwanese descent)
- ☐ Indigenous (e.g., First Nations, Inuk/Inuit, Metis descent)
- ☐ Latin America (e.g., Hispanic or Latin American descent)
- ☐ Middle Eastern (e.g., Arab, Persian, West Asian descent (e.g., Afghan, Egyptian, Iranian, Kurdish, Lebanese, Turkish))
- ☐ South Asian (e.g., Bangladeshi, Indian, Indo-Caribbean, Pakistani, Ski Lankan)
- ☐ Southeast Asian (e.g., Cambodian, Filipino, Indonesian, Thai, Vietnamese, or other Southeast Asian descent)
- ☐ White European
- ☐ White North American
- ☐ Other, please specify

____

Please indicate the highest education you have obtained

- ◯ Incomplete high school
- ◯ Completed high school
- ◯ College
- ◯ University
- ◯ Other, please specify

____

What is your age as of your last birthday (in years)?

____

Please indicate your total work experience in years.

Total years:

____

Total years as electrician:

____

Total years in Ontario:	__________________________________
In which position are you working? Please check all that apply	◯ Electrical sector<br>◯ Plumbing sector
Electrical sector	◯ Licensed electrician<br>◯ Electrical apprentices<br>◯ Electrical technician<br>◯ Electrical foreperson<br>◯ Maintenance technician<br>◯ Electrician journeyperson<br>◯ Electrical power supply technician<br>◯ Other, please specify in box below
	__________________________________
Plumbing sector	◯ Licensed plumber<br>◯ Plumbing apprentice<br>◯ Licensed journeyperson plumber<br>◯ Service technician<br>◯ Other, please specify in box below
	__________________________________
Please indicate the average number of hours you work per week	__________________________________
Do you intend to stay in your current position for the next 5 years?	◯ Yes<br>◯ No
If you selected "No", kindly specify the reason(s) why you do not anticipate staying (e.g., retirement):	__________________________________
How satisfied would you say you are with your job?	◯ I am very satisfied<br>◯ I am somewhat satisfied<br>◯ I am not too satisfied<br>◯ I am not satisfied at all
Please indicate the community where your workplace is located. (It would be helpful if you could provide both the community's name and postal code)	__________________________________
Optional: Were you diagnosed by a doctor or nurse practitioner with any chronic illness/physical injury?	◯ Yes<br>◯ No
Please specify below	__________________________________
When were you diagnosed with this illness/injury?	__________________________________
Height (feet and inches):	__________________________________

Weight (pounds): ______________________

Check the box that applied most to you

	Never/almost never or to a very low degree	Seldom or to a low degree	Sometimes or somewhat	Often or to a high degree	Always or to a very high degree
How often do you feel tired?	◯	◯	◯	◯	◯
How often are you physically exhausted?	◯	◯	◯	◯	◯
How often are you emotionally exhausted?	◯	◯	◯	◯	◯
How often do you think: "I can't take it anymore"?	◯	◯	◯	◯	◯
How often do you feel worn out?	◯	◯	◯	◯	◯
How often do you feel weak and susceptible to illness?	◯	◯	◯	◯	◯

Check the box that applied most to you

	Never/almost never or to a very low degree	Seldom or to a low degree	Sometimes or somewhat	Often or to a high degree	Always or to a very high degree
Do you feel worn out at the end of the working day?	◯	◯	◯	◯	◯
Are you exhausted in the morning at the thoughts of another day at work?	◯	◯	◯	◯	◯
Do you feel that every working hour is tiring for you?	◯	◯	◯	◯	◯
Do you have enough energy for family and friends during leisure time?	◯	◯	◯	◯	◯
Is your work emotionally exhausting?	◯	◯	◯	◯	◯
Does your work frustrate you?	◯	◯	◯	◯	◯
Do you feel burnt out because of your work?	◯	◯	◯	◯	◯

Have you at any time during the last 12 months had trouble (ache, pain, discomfort, numbness) in:

Neck
◯ No
◯ Yes

Have you at any time during the last 12 months been prevented from doing your normal work (at home or away from home) because of the trouble?
◯ No
◯ Yes

Have you had this trouble at any time during the last 7 days:	◯ No<br>◯ Yes
Shoulders	◯ No<br>◯ Yes, right shoulder<br>◯ Yes, left shoulder<br>◯ Yes, both shoulders
Have you at any time during the last 12 months been prevented from doing your normal work (at home or away from home) because of the trouble?	◯ No<br>◯ Yes
Have you had this trouble at any time during the last 7 days:	◯ No<br>◯ Yes
Elbows	◯ No<br>◯ Yes, right elbow<br>◯ Yes, left elbow<br>◯ Yes, both elbows
Have you at any time during the last 12 months been prevented from doing your normal work (at home or away from home) because of the trouble?	◯ No<br>◯ Yes
Have you had trouble at any time during the last 7 days:	◯ No<br>◯ Yes
Wrists/hands	◯ No<br>◯ Yes, right wrist/hand<br>◯ Yes, left wrist/hand<br>◯ Yes, both wrist/hand
Have you at any time during the last 12 months been prevented from doing your normal work (at home or away from home) because of the trouble?	◯ No<br>◯ Yes
Have you had this trouble at any time during the last 7 days:	◯ No<br>◯ Yes
Upper back	◯ No<br>◯ Yes
Have you at any time during the last 12 months been prevented from doing your normal work (at home or away from home) because of the trouble?	◯ No<br>◯ Yes
Have you had this trouble at any time during the last 7 days:	◯ No<br>◯ Yes
Lower back	◯ No<br>◯ Yes
Have you at any time during the last 12 months been prevented from doing your normal work (at home or away from home) because of the trouble?	◯ No<br>◯ Yes
Have you had this trouble at any time during the last 7 days:	◯ No<br>◯ Yes

One or both hips	◯ No<br>◯ Yes
Have you at any time during the last 12 months been prevented from doing your normal work (at home or away from home) because of the trouble?	◯ No<br>◯ Yes
Have you had this trouble at any time during the last 7 days:	◯ No<br>◯ Yes
One or both knees	◯ No<br>◯ Yes
Have you at any time during the last 12 months been prevented from doing your normal work (at home or away from home) because of the trouble?	◯ No<br>◯ Yes
Have you had this trouble at any time during the last 7 days:	◯ No<br>◯ Yes
One or both ankles/feet	◯ No<br>◯ Yes
Have you at any time during the last 12 months been prevented from doing your normal work (at home or away from home) because of the trouble?	◯ No<br>◯ Yes
Have you had trouble at any time during the last 7 days:	◯ No<br>◯ Yes
In general, would you say your health is:	◯ Excellent<br>◯ Very Good<br>◯ Good<br>◯ Fair<br>◯ Poor

The following two questions are about activities you might do during a typical day. Does YOUR HEALTH NOW LIMIT YOU in these activities? If so, how much?

MODERATE ACTIVITIES, such as moving a table, pushing a vacuum cleaner, bowling, or playing golf:	◯ Yes, Limited A Lot<br>◯ Yes, Limited A Little<br>◯ No, Not Limited At All
Climbing SEVERAL flights of stairs:	◯ Yes, Limited A Lot<br>◯ Yes, Limited A Little<br>◯ No, Not Limited At All

During the PAST MONTH have you had any of the following problems with your work or other regular activities AS A RESULT OF YOUR PHYSICAL HEALTH?

ACCOMPLISHED LESS than you would like:	◯ Yes<br>◯ No
Were limited in the KIND of work or other activities?	◯ Yes<br>◯ No

During the PAST MONTH, were you limited in the kind of work you do or other regular activities AS A RESULT OF ANY EMOTIONAL PROBLEMS (such as feeling depressed or anxious)?

ACCOMPLISHED Less than you would like:	◯ Yes<br>◯ No
Didn't do work or other activities as CAREFULLY as usual:	◯ Yes<br>◯ No
During the PAST MONTH, how much did PAIN interfere with your normal work (including both work outside the home and housework)?	◯ Not at all<br>◯ A little bit<br>◯ Moderately<br>◯ Quite a bit<br>◯ Extremely

The next three questions are about how you feel and how things have been DURING THE PAST MONTH. For each question, please give the one answer that comes closest to the way you have been feeling. How much of the time during the PAST MONTH

Have you felt calm and peaceful?	◯ All of the time<br>◯ Most of the time<br>◯ A good bit of the time<br>◯ Some of the time<br>◯ A little of the time<br>◯ None of the time
Did you have a lot of energy?	◯ All of the time<br>◯ Most of the time<br>◯ A good bit of the time<br>◯ Some of the time<br>◯ A little of the time<br>◯ None of the time
Have you felt downhearted and blue?	◯ All of the time<br>◯ Most of the time<br>◯ A good bit of the time<br>◯ Some of the time<br>◯ A little of the time<br>◯ None of the time
During the PAST MONTH, how much of the time has your PHYSICAL HEALTH OR EMOTIONAL PROBLEMS interfered with your social activities (like visiting with friends, relatives, etc.)?	◯ All of the Time<br>◯ Most of the Time<br>◯ A Good Bit of the Time<br>◯ Some of the Time<br>◯ A Little of the Time<br>◯ None of the Time
During the past month, what time have you usually gone to bed at night?	________________________
During the past month, how long (in minutes) has it usually taken you to fall asleep each night?	________________________
During the past month, what time have you usually gotten up in the morning?	________________________
During the past month, how many hours of actual sleep did you get at night? (This may be different than the number of hours you spend in bed).	________________________

For each of the remaining questions, check one best response. Please answer all questions...
During the past month, how often have you had trouble sleeping because you

	Not during the past month	Less than once a week	Once or twice a week	Three or more times a week
Cannot get sleep within 30 minutes	◯	◯	◯	◯
Wake up in the middle of the night or early morning	◯	◯	◯	◯
Have to get up to use the bathroom	◯	◯	◯	◯
Cannot breathe comfortably	◯	◯	◯	◯
Cough or snore loudly	◯	◯	◯	◯
Feel too cold	◯	◯	◯	◯
Feel too hot	◯	◯	◯	◯
Had bad dreams	◯	◯	◯	◯
Have pain	◯	◯	◯	◯

Other Reason(s). Please describe ______________________________

How often during the past month have you had trouble sleeping because of this?

◯ Not during the past month
◯ Less than once a week
◯ Once or twice a week
◯ Three or more times a week

During the past month, how would you rate your overall sleep quality?

◯ Very good
◯ Fairly good
◯ Fairly bad
◯ Very bad

During the past month,

	Not during the past month	Less than once a week	Once or twice a week	Three or more times a week
How often have you taken medicine to help you sleep (prescribed or "over the counter")?	◯	◯	◯	◯
How often have you had trouble staying awake while driving, eating meals, or engaging in social activity?	◯	◯	◯	◯
How much of a problem has it been for you to keep up enough enthusiasm to get things done?	◯	◯	◯	◯

Do you have a bedroom partner or roommate?

- ◯ No bed partner or roommate
- ◯ Partner/roommate in other room
- ◯ Partner in same room, but not same bed
- ◯ Partner in same bed

For each of the remaining questions, check one best response. Please answer all questions. If you have a roommate or a bed partner, ask him/her how often in the past you have...

	Not during the past month	Less than once a week	Once or twice a week	Three or more times a week
Loud snoring	◯	◯	◯	◯
Long pauses between breaths while sleeping	◯	◯	◯	◯
Legs twitching or jerking while you sleep	◯	◯	◯	◯
Episodes of disorientation or confusion during sleep	◯	◯	◯	◯

How often during the past month have you had trouble sleeping because of your bedroom partner or roommate?

- ◯ Not during the past month
- ◯ Less than once a week
- ◯ Once or twice a week
- ◯ Three or more times a week

The following questions ask about how you have been feeling during the past month:

	All of the time	Most of the time	Some of the time	A little of the time	None of the time
Nervous?	◯	◯	◯	◯	◯
Hopeless?	◯	◯	◯	◯	◯
Restless or fidgety?	◯	◯	◯	◯	◯
So depressed that nothing could cheer you up?	◯	◯	◯	◯	◯

That everything was an effort?	◯	◯	◯	◯	◯
Worthless?	◯	◯	◯	◯	◯

Emergency and Crisis Lines
If you are experiencing long-term stress and/or imminent health and safety risk, please consider reaching out to a healthcare professional or emergency services. Please access the following link for a list of emergency crisis lines and additional resources:

[Attachment: "Mental Health Resource List.pdf"]
